# Healthcare resource utilization and cost burden of COVID-19 according to vaccination status in adults in Ontario, Canada, 2021–2023

**DOI:** 10.1371/journal.pone.0344690

**Published:** 2026-04-22

**Authors:** Chloe McDonald, Ana Gabriela Grajales, Nassim Ait Yahia, David Fisman, Irene-Yanran Wang, Natalie Nightingale, Calum S. Neish, Saranya Nair, Mustapha Mustapha, Jingyan Yang

**Affiliations:** 1 Medical Affairs, Pfizer Canada, Kirkland, QC, Canada; 2 Market Access, Pfizer Canada, Kirkland, QC, Canada; 3 Dalla Lana School of Public Health, University of Toronto, Toronto, ON, Canada; 4 Real World Solutions, IQVIA Solutions Canada Inc, Mississauga, QC, Canada; 5 Global Value and Evidence, COVID Vaccine, Pfizer Inc. New York, New York, United States of America; Independent Medical Researcher and Writer, UNITED KINGDOM OF GREAT BRITAIN AND NORTHERN IRELAND

## Abstract

**Aims:**

This study aims to describe healthcare resource utilization (HCRU) and cost burden associated with COVID-19 cases in Ontario, Canada, and assess variations by vaccination status.

**Methods:**

We conducted a population-based retrospective cohort study using administrative health data from Ontario, focusing on COVID-19 cases from January 1, 2021, to May 31, 2023. Cases were identified by their first positive PCR test, and their healthcare interactions were followed for up to 12 months post-infection. All-cause HCRU and direct healthcare cost were compared before and after infection using generalized linear models.

**Results:**

Data from a total of 1,321,174 COVID-19 cases were analyzed, with 87% having at least one healthcare interaction in the year preceding their infection. All-cause HCRU increased post-infection, with the greatest rise observed in the first month, largely attributable to hospitalizations (Mean [SD] hospitalizations per person per month [PPPM] at Month 1 vs. Look-back: 1.1 [0.3] vs. 0.1 [0.1], p < .0001) and ICU admissions (mean [SD] admissions PPPM at Month 1 vs. Look-back: 1.0 [0.1] vs. 0.1 [0.0], p < .0001). The healthcare cost per person rose in both the six and twelve months pre- versus post-infection. This increase is primarily attributable to ICU admission, hospitalization, and mechanical ventilation costs. On average, vaccinated individuals exhibited smaller increases in ICU costs (vaccinated: + CAD 5,838 to CAD 18,730 vs. unvaccinated: + CAD 24,597) and hospitalization costs (vaccinated: + CAD 623 to CAD 5,201 vs. unvaccinated: + CAD 9,072) six months post-infection compared to unvaccinated cases.

**Conclusions:**

Data from this study cohort suggests that COVID-19 strains healthcare resources, namely as a result of hospitalization and mechanical ventilation. Vaccinated individuals demonstrated a reduced burden compared to their unvaccinated counterparts. These findings underscore the importance of vaccination in reducing severe outcomes and healthcare costs, providing insights for public health strategies and individual patient counseling.

## Introduction

Since the onset of the COVID-19 pandemic, Canada has reported a total of 4,946,090 cases and 59,034 deaths due to COVID-19 [1, 2]. About 10% of COVID-19 patients in Canada required hospitalization, with 22% of those hospitalized needing ICU care resulting in an average ICU stay of 20.5 days for patients on invasive mechanical ventilation [3]. Additionally, the occurrence of death and unplanned readmission within 30 days post-discharge have been common, with one in nine discharged patients experiencing either death or readmission within this period [4]. Therefore, populations, including those with comorbidities, immunocompromised conditions, and residents of long-term care (LTC) facilities, face an increased risk of severe outcomes following COVID-19 [5].

Economically, the pandemic has imposed substantial costs on the healthcare system. Direct costs encompass expenses related to testing and clinical management, while indirect costs arise from the long-term care required for patients with post-COVID-19 conditions (PCC). Estimates suggest that the total cost of COVID-19 hospital stays in Canada from April 2022 to March 2023 was approximately $4.1 billion and the average cost per COVID-19 hospital stay was estimated at $24,400, roughly three times higher than the cost of an average hospital stay [6].

Understanding the healthcare resource utilization (HCRU) and subsequent costs for COVID-19 cases, while exploring variations based on vaccination history, is crucial for optimizing healthcare strategies and resource allocation, ultimately improving patient outcomes and reducing financial burdens on the healthcare system. In Canada, comprehensive public health measures, including the COVID-19 vaccination program, have effectively reduced infection rates and, consequently, healthcare demand [7, 8]. By mid-May 2021, 50% of Canadians had received at least one dose [9], a figure that increased to 81.1% in July 2024 [10]. However, vaccine-induced antibodies wane approximately 6 months after the primary vaccination series (i.e., two doses of a two-dose vaccine or one dose of a one-dose vaccine), and vaccine effectiveness against infections and hospitalizations may also decrease 2–7 months post-vaccination in absence of booster doses [11, 12]. These outcomes also vary by SARS-CoV-2 strain, highlighting the need to specify strain context when evaluating vaccine performance and related health impacts. Therefore, this study aims to describe all-cause HCRU and costs among incident PCR-positive COVID-19 cases in Ontario, Canada between January 1, 2021 and May 31, 2023, and explores variations in these outcomes by vaccination status and time since last vaccine dose.

## Materials and methods

### Study design, population and period

This population-based retrospective cohort study utilized administrative health data from Ontario, Canada, the country’s most populous province with approximately 15.9 million residents. Ontario’s single-payer, publicly funded healthcare system ensures that the administrative data used in this study represents near-census coverage of Ontarian residents.

The study focused on PCR-positive COVID-19 cases. Cases were identified by their first positive PCR test for SARS-CoV-2 (i.e., the index date) within the selection period from January 1, 2021, to May 31, 2023, spanning both the pre-Omicron variant period (January to December 2021) and the Omicron variant-dominant period (mid-December 2021 to May 2023). To be included in the study, PCR-positive COVID-19 cases also needed to be at least 6 months old on the index date. Individuals were excluded from the study if they had an invalid Ontario Health Insurance Plan (OHIP) card number or were not OHIP-eligible on the index date. Those with invalid or incomplete records, such as missing age, sex, or a death date prior to the index, were not included. People who were not Ontario residents on the index date were excluded as well. Anyone aged 105 years or older at the time of the index date was not eligible, and individuals who were OHIP eligible for less than one month before the index date were also excluded from the study.

PCR-positive COVID-19 cases were followed from their index date (inclusive) for up to 12 months or until a censoring event occurred. Censoring events included death, new COVID-19 infection (defined as a positive COVID-19 PCR test more than 90 days after the index date), loss to follow-up in the database, end of OHIP coverage, or the conclusion of data availability on June 30, 2023. Additionally, cases were observed for a minimum of one month and a maximum of 12 months prior to the index date, referred to as “the preceding year”, to assess their HRCU and direct healthcare costs leading up to the index.

### Data source

This study linked multiple healthcare administrative databases held at ICES (Institute for Clinical Evaluative Sciences) to identify the population of interest and operationalize outcomes. ICES is an independent, not-for-profit research institute that, under Ontario’s health information privacy law, is authorized to collect and analyze healthcare data for health system evaluation and improvement. The OHIP is a single payer healthcare insurance, which provides medically necessary services to all the residents of Ontario. Patients’ encrypted OHIP card number was used to link the ICES datasets to follow the patient through each healthcare touchpoint. All data sources were integrated at the patient level to allow for patient-level, longitudinal analysis. The Ontario Laboratory Information System COVID-19 subset (OLIS-C19) was employed to identify individuals with a positive COVID-19 infection based on lab test orders and results from hospitals, community labs, and public health labs. Information regarding COVID-19 vaccinations was sourced from COVaxON, a central registry that maintains comprehensive records of all COVID-19 vaccinations administered in Ontario. The vaccination program commenced on December 14, 2020, with the majority of residents receiving mRNA-based vaccines, such as Moderna Spikevax and Pfizer-BioNTech Comirnaty. The presence of comorbidities was assessed using validated algorithms derived from diagnostic and procedure codes across multiple databases. Further details about these databases and the associated variables can be found in the S2 Table. The ICES datasets were accessed between 11 January and 31 July 2024. Authors received only aggregated results and had no access to transaction-level data.

### Exposures and outcomes of interest

All PCR-positive COVID-19 cases meeting the eligibility criteria described above were included in the HCRU analysis, forming the ***HCRU Cohort***. Additionally, a subset of these cases, with a minimum of 6 months of follow-up from the study index to the conclusion of cost data availability on March 31, 2023 (i.e., indexed before September 30, 2022), were selected for direct health cost analysis. These cases make up the ***Direct Cost Cohort*** and were followed for either 6 months or 12 months from the index date (inclusive).

Among PCR-positive COVID-19 cases, the COVID-19 vaccination status was evaluated as a key exposure of interest. The dataset comprised variables related to patient demographics, clinical characteristics (e.g., COVID-19 vaccination status, time since last positive PCR test for SARS-CoV-2, comorbidities, among others.), treatments, all-cause HCRU, and direct healthcare costs. These variables are detailed in S3 Table. The outcomes measured included all-cause HCRU measurements (e.g., physician visits, emergency department (ED) visits, inpatient hospitalizations, length of hospital stay, duration in the intensive care unit (ICU), mechanical ventilation use) and all-cause direct healthcare costs.

### Data analysis

Descriptive statistics were used to summarize continuous data, including the number of observations (N), mean, standard deviation (SD), median, first and third quartiles, as well as minimum and maximum values. Categorical and ordinal data were summarized using frequencies and percentages. All-cause HCRU was calculated as encounters per person per month (PPPM) in the preceding year and number of encounters per patient for each month following the index date. Direct healthcare costs were calculated for the sub-population and aggregated over periods of up to 6 months or 12 months before and after the index date. These costs were estimated over 6-month and 12-month periods pre- versus post-index, versus monthly estimation of all-cause HCRU, as cost estimates are derived from multiple data sources and can only be estimated with validity over periods of at least 6 months. The hospital-based costs were calculated using the resource intensity weight methodology [13] from the Canadian Management Information System (MIS) Database, which attributes a hospital specific cost to the resource intensity of each visit. All costs were standardized to 2021 Canadian dollars (CAD$).

To compare mean differences in all-cause HCRU and direct costs before and after COVID-19 infection, generalized linear models were calculated. For all-cause HCRU, p-values from Poisson or Negative Binomial models were calculated to compare counts data. For direct costs, an unadjusted Gamma model was applied, using the appropriate link function (either canonical inverse power or log link, depending on convergence) to assess variance and compare groups. Mean difference along with 95% confidence intervals (CI) and p-values were reported from each model.

All outcome variables were stratified by COVID-19 vaccination status (none, initiated primary series, completed primary series, completed primary series and up-to-date vaccine) and days since last COVID-19 vaccine dose (<14, 14–89, 90–179, 180–269, ≥ 270 days, no prior vaccine dose). Variable and category definitions for stratified analyses are provided in the supplementary materials.

Any groups smaller than 6 individuals were suppressed, along with the second smallest group to prevent back-calculation (i.e., double suppression). Analyses were conducted using Statistical Analysis System (SAS) version 9.3 or higher (SAS Institute Inc., Cary, NC, United States).

### Ethics

This study was designed and implemented with ethics approval from the Institutional Review Board Services (Advarra IRB# 00000971, Approval #Pro00075923) and was approved by the ICES Privacy and Compliance Office. The ethics committee waived the requirement for informed consent.

## Results

A total of 1,321,174 cases with a positive SARS-CoV-2 PCR result aged 6 months or older were identified in 2021 (n = 627,973), 2022 (n = 639,080), and between January 1 and May 31 in 2023 (n = 54,121) in Ontario. The median age of these cases was 40 years, with 55.8% being female, and 84.1% concentrated in large urban areas. Among the PCR-positive COVID-19 cases, 52,981 (4.0%) were residents of a long-term care facility (LTC) at index (Table 1). The most prevalent pre-existing comorbidities among PCR-positive COVID-19 cases were hypertension (19.8%), chronic respiratory disease (13.4%), and dementia (11.2%) (Table 2).

**Table 1:** Demographic characteristics for COVID-19 cases.

	HCRU cohort	Direct cost cohort
Number of cases	N = 1,321,174	N = 1,189,005
Year of index date		
2021	627,973 (47.5%)	627,973 (52.8%)
2022	639,080 (48.4%)	561,032 (47.2%)
2023	54,121 (4.1%)	0 (0.0%)
Age (in years)		
Mean, SD	43.1 (23.1)	41.0 (22.1)
Median (IQR)	40 (26-59)	38 (25-56)
Min – Max	0 - 104	0 - 104
Age Group (in years)		
0.5 to 4	36,002 (2.7%)	34,051 (2.9%)
5–11	69,506 (5.3%)	68,466 (5.8%)
12–17	62,831 (4.8%)	61,668 (5.2%)
18–64	905,485 (68.5%)	843,724 (71.0%)
≥ 65	247,350 (18.7%)	181,096 (15.2%)
Genotypic Sex		
Female	736,699 (55.8%)	653,371 (55.0%)
Male	584,475 (44.2%)	535,634 (45.0%)
Rurality		
Missing	4,205 (0.3%)	3,442 (0.3%)
Rural	113,384 (8.6%)	96,991 (8.2%)
Medium urban	92,225 (7.0%)	78,041 (6.6%)
Large urban	1,111,360 (84.1%)	1,010,531 (85.0%)
Local Health Integration Network		
Erie St. Clair	66,790 (5.1%)	59,493 (5.0%)
South West	78,705 (6.0%)	68,449 (5.8%)
Waterloo Wellington	66,435 (5.0%)	59,624 (5.0%)
Hamilton Niagara Haldimand Brant	148,387 (11.2%)	131,936 (11.1%)
Central West	110,230 (8.3%)	104,054 (8.8%)
Mississauga Halton	112,573 (8.5%)	104,071 (8.8%)
Toronto Central	128,005 (9.7%)	118,213 (9.9%)
Central	184,912 (14.0%)	170,358 (14.3%)
Central East	147,560 (11.2%)	132,976 (11.2%)
South East	45,637 (3.5%)	38,203 (3.2%)
Champlain	104,767 (7.9%)	93,273 (7.8%)
North Simcoe Muskoka	44,234 (3.3%)	38,622 (3.2%)
North East	53,123 (4.0%)	44,628 (3.8%)
North West	29,816 (2.3%)	25,105 (2.1%)
Neighbourhood income quintile		
Missing	4,671 (0.4%)	3,867 (0.3%)
Q1, lowest	284,227 (21.5%)	250,706 (21.1%)
Q2	262,622 (19.9%)	234,028 (19.7%)
Q3	266,192 (20.1%)	241,496 (20.3%)
Q4	256,286 (19.4%)	232,388 (19.5%)
Q5, highest	247,176 (18.7%)	226,520 (19.1%)
Long-term care residency	52,981 (4.0%)	35,987 (3.0%)

**Table 2:** Clinical Characteristics for COVID-19 cases.

	HCRU cohort	Direct cost cohort
Number of cases	N = 1,321,174	N = 1,189,005
**Comorbidities**		
*Chronic heart disease*	103,834 (7.9%)	75,302 (6.3%)
Cardiac ischemic disease	47,075 (3.6%)	34,968 (2.9%)
Atrial fibrillation	46,924 (3.6%)	33,468 (2.8%)
Chronic heart failure	53,207 (7.8%)	36,859 (6.4%)
*Chronic respiratory disease*	176,452 (13.4%)	151,210 (12.7%)
Asthma	113,384 (8.6%)	103,073 (8.7%)
Chronic obstructive pulmonary disease	76,368 (9.8%)	58,378 (8.7%)
*Hypertension*	222,232 (19.8%)	181,590 (18.2%)
*Diabetes*	137,120 (10.4%)	112,492 (9.5%)
*Immunocompromised status*	109,417 (8.3%)	85,847 (7.2%)
Transplant recipient	7,066 (0.5%)	5,380 (0.5%)
Sickle-cell disease	610 (0.0%)	543 (0.0%)
Other immune system disorders	23,784 (1.8%)	19,307 (1.6%)
Cancer	82,058 (6.2%)	63,309 (5.3%)
Active Cancer	21,759 (1.6%)	16,038 (1.3%)
Human immunodeficiency virus	2,021 (0.2%)	1,831 (0.2%)
*Autoimmune disease*	40,819 (3.1%)	34,231 (2.9%)
Inflammatory bowel disease	6,815 (0.5%)	5,985 (0.5%)
Psoriasis/psoriatic arthritis	17,453 (1.3%)	14,910 (1.3%)
Multiple sclerosis	4,254 (0.3%)	3,365 (0.3%)
Rheumatoid arthritis	13,846 (1.2%)	11,209 (1.1%)
*Chronic kidney disease*	59,331 (4.5%)	43,551 (3.7%)
*Advanced liver disease*	10,817 (0.8%)	8,379 (0.7%)
*Stroke*	20,318 (1.5%)	14,107 (1.2%)
*Obesity*	24,937 (1.9%)	20,370 (1.7%)
*Down’s syndrome*	903 (0.1%)	741 (0.1%)
*Pregnancy*	23,402 (2.6%)	21,238 (2.5%)
*Dementia*	75,801 (11.2%)	51,550 (9.0%)
**Prior COVID-19 vaccination**		
*Number of doses*		
0	442,597 (33.5%)	432,655 (36.4%)
1	68,692 (5.2%)	67,207 (5.7%)
2	377,593 (28.6%)	361,362 (30.4%)
≥ 3	432,292 (32.7%)	327,781 (27.6%)
*Vaccination status*		
initiated series	68,017 (5.1%)	66,565 (5.6%)
completed primary series	229,766 (17.4%)	214,560 (18.0%)
completed primary series + updated dose	580,794 (44.0%)	475,225 (40.0%)
*Type of COVID-19 vaccine*		
mRNA	832,483 (63.0%)	714,519 (60.1%)
non-mRNA	9,186 (0.7%)	8,938 (0.8%)
combination of mRNA and non-mRNA	36,908 (2.8%)	32,893 (2.8%)
*Time since last vaccine dose, days*		
Mean, SD	153.3 (109.2)	137.2 (88.4)
Median (IQR)	152 (70-201)	144 (64-190)
Min – Max	1 - 836	1 - 626
< 14 days	70,182 (5.3%)	66,008 (5.6%)
14–89 days	197,450 (14.9%)	177,652 (14.9%)
90–179 days	294,268 (22.3%)	270,695 (22.8%)
180–269 days	209,701 (15.9%)	193,974 (16.3%)
≥ 270 days	106,976 (8.1%)	48,021 (4.0%)
*Time since last positive PCR test for SARS-CoV-2, days*		
Any, n (%)	17,986 (1.4%)	14,534 (1.2%)
Mean, SD	504.6 (275.0)	424.1 (237.5)
Median (IQR)	515 (381-698)	447 (344-604)
Min – Max	1 - 1162	1 - 923
< 14 days	697 (0.1%)	697 (0.1%)
14–89 days	1,895 (0.1%)	1,895 (0.2%)
90–179 days	637 (0.0%)	637 (0.1%)
180–269 days	234 (0.0%)	234 (0.0%)
≥ 270 days	14,523 (1.1%)	11,071 (0.9%)

Two thirds (66.5%) of cases were recently vaccinated an average (SD) of 153 (109.2) days before their index COVID-19 infection, while the remaining third had no record of prior COVID-19 vaccination (Table 2). Among the vaccinated, 92.3% completed at least a primary vaccination series, and 66.1% received at least one vaccine update (i.e., an additional dose). Notably, 99.0% of vaccinated PCR-positive COVID-19 cases received at least one mRNA vaccine, including those who received a combination of mRNA and non-mRNA vaccines. Furthermore, 98.6% of PCR-positive COVID-19 cases had no prior positive PCR test for SARS-CoV-2. Among the 17,986 cases with a previous infection, the mean (SD) time since their last positive PCR test was 505 (275.0) days (Table 2).

Cases in the direct cost cohort (i.e., those infected between January 1, 2021, and September 30, 2022) exhibited demographic and clinical characteristics similar to those in the HCRU cohort (Table 1,Table 2).

### Hospitalizations and ICU admission

Overall, 87% of PCR-positive COVID-19 cases had a minimum of one interaction with the healthcare system in the year leading up to their index COVID-19 infection, with this proportion increasing slightly to 91% in the year following the infection (Table 3).

**Table 3:** Healthcare Resource Utilization in the Lookback Period and Months 1-3, HCRU cohort.

	Lookback Period (PPPM)	Month 1	Month 2	Month 3
Number of cases	N = 1,321,174	N = 1,321,174	N = 1,301,754	N = 1,292,007
Total person months follow-up	15,840,474	1,309,904	1,296,782	1,287,651
**General Practitioner (GP) visits at any settings**				
Any – n (%)	1,058,467 (80.1%)	614,468 (46.5%)	407,297 (31.3%)	384,233 (29.7%)
Mean (SD)	0.7 (0.9)	2.7 (3.6)	2.2 (3.1)	2.0 (2.5)
Median (Q1-Q3)	0 (0-1)	1 (1 –3)	1 (1 –2)	1 (1 –2)
Min – Max	0 - 29	1 - 51	1 - 49	1 - 47
**Specialists visits at any settings**				
Any – n (%)	897,233 (67.9%)	431,607 (32.7%)	323,412 (24.8%)	304,844 (23.6%)
Mean (SD)	0.9 (1.8)	5.5 (9.9)	3.4 (6.9)	3.0 (5.6)
Median (Q1-Q3)	0 (0-1)	2 (1 –5)	1 (1 –3)	1 (1 –3)
Min – Max	0 - 89	1 - 277	1 - 187	1 - 204
**Emergency department visits**				
Any – n (%)	401,739 (30.4%)	171,290 (13.0%)	54,402 (4.2%)	51,703 (4.0%)
Mean (SD)	0.2 (0.2)	1.2 (0.6)	1.2 (0.8)	1.2 (0.8)
Median (Q1-Q3)	0 (0−0)	1 (1 –1)	1 (1 –1)	1 (1 –1)
Min – Max	0 - 30	1 - 29	1 - 30	1 - 30
**npatient Hospitalization**				
Any – n (%)	130,469 (9.9%)	88,125 (6.7%)	18,177 (1.4%)	15,682 (1.2%)
Mean (SD)	0.1 (0.1)	1.1 (0.3)	1.1 (0.3)	1.1 (0.3)
Median (Q1-Q3)	0 (0−0)	1 (1 –1)	1 (1 –1)	1 (1 –1)
Min – Max	0 - 3	1 - 7	1 - 6	1 - 10
**ICU admission**				
Any – n (%)	20,682 (1.6%)	15,655 (1.2%)	2,169 (0.2%)	1,731 (0.1%)
Mean (SD)	0.1 (0.0)	1.0 (0.1)	1.0 (0.1)	1.0 (0.2)
Median (Q1-Q3)	0 (0−0)	1 (1 –1)	1 (1 –1)	1 (1 –1)
Min – Max	0 - 2	1 - 3	1 - 2	1 - 3
**Time spent in hospital, days**				
Any – n (%)	130,469 (9.9%)	88,125 (6.7%)	18,177 (1.4%)	15,682 (1.2%)
Mean (SD)	1.5 (2.3)	9.7 (8.0)	7.5 (6.7)	7.1 (6.3)
Median (Q1-Q3)	1 (0-2)	7 (3 –13)	5 (3 –10)	5 (3 –9)
Min – Max	0 - 30	1 - 30	1 - 30	1 - 30
**Time spent in ICU, days**				
Any – n (%)	20,682 (1.6%)	15,655 (1.2%)	2,169 (0.2%)	1,731 (0.1%)
Mean (SD)	0.7 (1.3)	9.1 (7.9)	5.8 (5.4)	5.2 (4.8)
Median (Q1-Q3)	0 (0-1)	6 (3 –13)	4 (2 –7)	4 (2 –6)
Min – Max	0 - 29	1 - 30	1 - 30	1 - 30
**Instances of mechanical ventilation use**				
Any – n (%)	7,456 (0.6%)	7,639 (0.6%)	848 (0.1%)	642 (0.0%)
Mean (SD)	0.1 (0.0)	1.0 (0.1)	1.0 (0.1)	1.0 (0.1)
Median (Q1-Q3)	0 (0−0)	1 (1 –1)	1 (1 –1)	1 (1 –1)
Min – Max	0 - 1	1 - 2	1 - 3	1 - 2
**Same-Day Surgery**				
Any – n (%)	94,203 (7.1%)	10,417 (0.8%)	12,277 (0.9%)	11,397 (0.9%)
Mean (SD)	0.1 (0.1)	1.1 (0.5)	1.1 (0.5)	1.1 (0.4)
Median (Q1-Q3)	0 (0−0)	1 (1 –1)	1 (1 –1)	1 (1 –1)
Min – Max	0 - 8	1 - 21	1 - 12	1 - 12
**Long-Term Care Admission**				
Any – n (%)	52,981 (4.0%)	54,321 (4.1%)	52,366 (4.0%)	52,095 (4.0%)
**Time spent in Long-Term Care, days**				
Any – n (%)	51,713 (3.9%)	53,132 (4.0%)	51,275 (3.9%)	51,069 (4.0%)
Mean (SD)	24.8 (9.0)	27.8 (6.4)	28.6 (5.0)	28.8 (4.6)
Median (Q1-Q3)	30 (22 –30)	30 (30 –30)	30 (30 –30)	30 (30 –30)
Min – Max	0 - 30	1 - 30	1 - 30	1 - 30
**Home Care Service**				
Any – n (%)	113,686 (8.6%)	76,939 (5.8%)	67,172 (5.2%)	60,908 (4.7%)
Mean (SD)	6.4 (8.9)	8.9 (9.3)	11.6 (10.6)	12.3 (10.9)
Median (Q1-Q3)	2 (0-9)	5 (2 –13)	7 (2-21)	8 (3-23)
Min – Max	0 - 30	1 - 30	1 - 30	1 - 30
**Inpatient Rehabilitation Service**				
Any – n (%)	7,828 (0.6%)	4,164 (0.3%)	1,354 (0.1%)	710 (0.1%)
Mean (SD)	0.1 (0.0)	1.0 (0.1)	1.0 (0.1)	1.0 (0.1)
Median (Q1-Q3)	0 (0−0)	1 (1 –1)	1 (1 –1)	1 (1 –1)
Min – Max	0 − 0	1 - 2	1 - 2	1 - 2
**Any HCRU (listed above)**				
Any – n (%)	1,149,800 (87.0%)	764,887 (57.9%)	570,819 (43.8%)	547,196 (42.4%)
Mean (SD)	2.0 (4.6)	6.7 (11.1)	5.1 (8.9)	4.6 (8.1)
Median (Q1-Q3)	1 (0-2)	2 (1 –6)	2 (1 –4)	2 (1 –4)
Min – Max	0 - 91	1 - 282	1 - 187	1 - 208

During the first month post-index infection (i.e., first positive PCR test for SARS-CoV-2 during the selection period), the number of hospitalizations per person was about 11 times higher than the per-person-per-month (PPPM) average in the preceding year (mean [SD]: 1.1 [0.3] vs. 0.1 [0.1], p < .0001) (Table 3; S2 File). The mean (SD) number of hospitalizations PPPM remained consistent at 1.1 (0.3) during the first three months after index. The proportion of cases experiencing at least one hospitalization was the highest in the first month (6.7%), dropping to 1.4% of cases thereafter (Table 3). The mean (SD) time spent in hospital per hospitalization was 9.7 (8.0) days in Month 1, 7.5 (6.7) days through Month 2, and 7.1 (6.3) days through Month 3, reflecting a 4- to 6-fold increase from the average time spent in hospital PPPM in the year preceding index (mean [SD]: 1.5 [2.3], p < .0001) (Table 3; S2 File).

A total of 15,655 (1.2%) PCR-positive COVID-19 cases were admitted to ICU in the first month following index, with an average (SD) of 9.1 (7.9) days spent in the ICU. That is, the number of ICU admissions among the cohort in just the first month following index is nearly equivalent to the total number of ICU admissions among the cohort in the entire year prior (20,682 cases, 1.6%). Further, the average ICU duration prior to index was significantly shorter, at 0.7 [1.3] days PPPM (p < .0001) (Table 3; S2 File). After Month 1, the proportion of cases with an ICU admission reduced to 0.2% in Month 2 then 0.1% in Month 3, while the average duration per admission shortened by about three to four days (Month 1: 9.1 [7.9] days; Month 2: 5.8 [5.4] days; Month 3: 5.2 [4.8] days) (Table 3).

A total of 7,639 (0.6%) PCR-positiveCOVID-19 cases required mechanical ventilation during at least one hospitalization in the first month after the index date. This proportion decreased by 88.7% in the second month, with only 848 (0.1%) cases experiencing a hospitalization requiring mechanical ventilation. The rate of hospitalizations requiring mechanical ventilation were the same in both months (mean [SD]: 1.1 [0.3]) (Table 3).

### ED visits

Thirteen percent of PCR-positive COVID-19 cases visited the ED at a mean (SD) rate of 1.2 (0.6) visits in the first month following index infection, representing a 6-fold increase compared to the PPPM average in the preceding year (mean [SD]: 0.2 [0.2], p < .0001) (Table 3; S2 File). The rate remained stable among the overall study population in the next two months post-index, despite a roughly 68% reduction in the proportion of cases visiting the ED (Table 3).

### Physician visits

Around half of cases (46.5%) had a visit to a general practitioner (GP) in any settings within a month of their index infection. This proportion decreased in the subsequent months (Month 2: 31.3%, Month 3: 29.7%). Following index infection, the mean (SD) numbers of GP visits were 2.7 (3.6) in Month 1, 2.2 (3.1) in Month 2, and 2.0 (2.5) in Month 3. This represents a significant increase compared to the PPPM average in the preceding year (mean [SD]: 0.7 [0.9], p < .0001) (Table 3; S2 File).

Similarly, specialist visits were most prevalent in the first month after index infection, observed in one-third (32.7%) of PCR-positive COVID-19 cases. Compared to the PPPM average in the preceding year (PPPM in the preceding year: 0.9 [1.8]; p < .0001) the mean (SD) number of specialist visits also increased significantly post index infection (Month 1: 5.5 [9.9], Month 2: 3.4 [6.9], Month 3: 3.0 [5.6] vs. PPPM in the preceding year: 0.9 [1.8]; p < .0001) (Table 3; S2 File).

### LTC and home care service

Overall, the number of PCR-positive COVID-19 cases residing in long-term care facilities remained stable, ranging from 52,000–55,000 both before and after index COVID-19 infection. The mean (SD) time spent in LTC increased from 24.8 (9.0) days PPPM in the preceding year to 27.8 (6.4) days in Month 1 post-index infection (Table 3).

The number of PCR-positive COVID-19 cases using home care services decreased after index infection compared to the preceding year, while the mean (SD) number of uses of homecare services increased through first three months post-index infection, doubling in Month 3 post-infection compared to the PPPM average in the preceding year (Month 1: 8.9 [9.3], Month 2: 11.6 [10.6], Month 3: 12.3 [10.9] vs. 6.4 [8.9], p < .0001) (Table 3; S2 File).

### Costs

Among cases in the Direct Cost Cohort, the total median (IQR) per-person healthcare cost increased in the 6 months following index infection compared to the respective period prior to index ($392 [$129-$1,680] vs. $316 [$110−41,308]). A greater increase was observed when excluding public drug plan costs ($361 [$123-$1,429] vs. $289 [$106-$1,088]) (Table 4; S2 File). The increase in total health care cost was primarily due to increased expenses of mechanical ventilation use ($69,622 [$33,287-$126,536] vs. $37,243 [$12,207-$85,971]), ICU ($33,287 [$15,524-$77,617] vs. $25,212 [$10,313-$53,497]), and hospitalization ($11,434 [$5,224-$22,814] vs. $8,403 [$3,822-$21,052]) (Table 4; S2 File).

**Table 4:** Direct Healthcare Cost in the Lookback Period and Analysis Period, Direct cost cohort.

	Up to 6 months pre-index date	Up to 6 months of follow-up	Up to 12 months pre-index date	Up to 12-months of follow-up
Number of cases	N = 1,189,005	N = 1,189,005	N = 1,189,005	N = 1,189,005
Total person months follow-up	7,134,030	7,006,075	14,256,747	13,703,878
Follow-up months				
Mean (SD)	6.0 (0.0)	5.9 (0.7)	12.0 (0.2)	11.5 (1.8)
Median (Q1-Q3)	6 (6 –6)	6 (6 –6)	12 (12 –12)	12 (12 –12)
Min – Max	6 − 6	0 - 6	6 - 12	0 - 12
Total costs				
Any – n (%)	1,102,741 (92.7%)	1,127,511 (94.8%)	1,133,211 (95.3%)	1,155,191 (97.2%)
Mean (SD)	$4278.6 ($15670.9)	$5872.9 ($20630.6)	$7278.7 ($24980.5)	$8733.1 ($28468.2)
Median (Q1-Q3)	$316 ($110-41308)	$392 ($129-$1680)	$664 ($230-$2524)	$808 ($276-$3283)
Min – Max	$1 - $757718	$1 - $916508	$1 - $1164143	$1 - $1246519
Total costs excluding public drug plan				
Any – n (%)	1,086,768 (91.4%)	1,111,913 (93.5%)	1,121,259 (94.3%)	1,143,386 (96.2%)
Mean (SD)	$3984.1 ($15221.8)	$5614.7 ($20310.3)	$6684.8 ($23939.0)	$8189.3 ($27565.5)
Median (Q1-Q3)	$289 ($106-$1088)	$361 ($123-$1429)	$611 ($221-$2120)	$751 ($266-$2775)
Min – Max	$1 - $757640	$1 - $916508	$1 - $1058966	$1 - $1246519
GP visits at any settings				
Any – n (%)	613,918 (51.6%)	678,988 (57.1%)	760,335 (63.9%)	826,452 (69.5%)
Mean (SD)	$242.2 ($624.1)	$271.0 ($556.8)	$355.0 ($950.4)	$373.0 ($814.9)
Median (Q1-Q3)	$103 ($41-$229)	$111 ($41-$261)	$144 ($52-$341)	$154 ($60-$370)
Min – Max	$1 - $162939	$1 - $55260	$1 - $262408	$1 - $126857
Specialists visits at any settings				
Any – n (%)	589,973 (49.6%)	659,024 (55.4%)	736,456 (61.9%)	803,868 (67.6%)
Mean (SD)	$753.3 ($1723.0)	$859.1 ($1956.2)	$1072.6 ($2298.8)	1149.6 ($2456.3)
Median (Q1-Q3)	$254 ($98-$672)	$272 ($107-$787)	$377 ($137-$1028)	403 (148-1150)
Min – Max	$1 - $76936	$1 - $91085	$1 - $98490	1 - 166263
Emergency department visits				
Any – n (%)	238,506 (20.1%)	280,795 (23.6%)	343,874 (28.9%)	384,321 (32.3%)
Mean (SD)	$761.6 ($1030.3)	$715.4 ($914.9)	$882.4 ($1429.2)	$821.2 ($1279.6)
Median (Q1-Q3)	$501 ($249-$923)	$470 ($255-$861)	$520 ($251-$1027)	$516 ($272-$953)
Min – Max	$67 - $110112	$67 - $106091	$67 - $204590	$67 - $205919
Inpatient Hospitalization				
Any – n (%)	66,452 (5.6%)	117,647 (9.9%)	97,465 (8.2%)	144,331 (12.1%)
Mean (SD)	$19255.6 ($31615.4)	$23063.1 ($38643.9)	$20509.6 ($36739.8)	$23398.0 ($42016.7)
Median (Q1-Q3)	$8403 ($3822-$21052)	$11434 ($5224-$22814)	$8202 ($3791-$21753)	$10942 ($4652-$22996)
Min – Max	$198 - $639596	$317 - $842796	$221 - $893422	$317 - $1097467
ICU^1^				
Any – n (%)	9,485 (0.80%)	17,874 (1.5%)	14,666 (0.78%)	22,235 (1.87%)
Mean (SD)	$43392.3 ($54537.3)	$59534.4 ($72160.4)	$48821.8 ($64831.5)	$61372.7 ($78526.8)
Median (Q1-Q3)	$25212 ($10313-$53497)	$33287 ($15524-$77617)	$26870 ($11113-$59918)	$33861 ($15987-$78255)
Min – Max	$269 - $639596	$539 - $842796	$269 - $893422	$539 - $1097467
Mechanical Ventilation Use^1^				
Any – n (%)	3,819 (0.32%)	8,395 (0.71%)	5,352 (0.45%)	9,953 (0.84%)
Mean (SD)	$62486.2 ($72964.5)	$93438.5 ($88663.2)	$74384.5 ($88549.6)	$95622.9 ($98457.1)
Median (Q1-Q3)	$37243 ($12207-$85971)	$69622 ($33287-$126536)	$43997 ($18103-$97831)	$68286 ($32831-$125737)
Min – Max	$269 - $639596	$765 - $842796	$269 - $893422	$1037 - $1097467
Same-Day Surgery				
Any – n (%)	45,055 (3.8%)	47,510 (4.0%)	78,016 (6.6%)	83,837 (7.1%)
Mean (SD)	$1867.3 ($2052.4)	$1970.0 ($2127.9)	$2027.8 ($2273.4)	$2076.3 ($2294.8)
Median (Q1-Q3)	$1011 ($731-$2416)	$1097 ($741-$2613)	$1208 ($784-$2620)	$1270 ($800-$2768)
Min – Max	$38 - $42016	$62 - $34627	$38 - $53756	$62 - $41520
Long-Term Care				
Any – n (%)	34,925 (2.9%)	40,775 (3.4%)	35,019 (2.9%)	43,058 (3.6%)
Mean (SD)	$24663.2 ($7637.1)	$22559.6 ($9614.9)	$44711.5 ($17831.6)	$39238.0 ($19608.1)
Median (Q1-Q3)	$26708 ($22641-$29245)	$25294 ($17559-$28963)	$49969 ($34762-$56670)	$44586 ($25156-$54538)
Min – Max	$86 - $47440	$86 - $48251	$86 - $94678	$86 - $96318
Home Care Service				
Any – n (%)	63,085 (5.3%)	74,767 (6.3%)	77,967 (6.6%)	86,060 (7.2%)
Mean (SD)	$4155.7 ($6721.6)	$3714.3 ($6247.8)	$6099.3 ($11236.6)	$5402.4 ($10564.5)
Median (Q1-Q3)	$1820 ($870-$4864)	$1481 ($778-$4137)	$2053 ($926-$6671)	$1596 ($778-$5477)
Min – Max	$20 - $211857	$10 - $188533	$19 - $323255	$10 - $385426
Complex Continuing Care				
Any – n (%)	4,331 (0.4%)	7,837 (0.7%)	5,790 (0.5%)	9,055 (0.8%)
Mean (SD)	$42226.7 ($38868.2)	$37398.4 ($36892.2)	$52445.0 ($60765.0)	$45237.1 ($56669.8)
Median (Q1-Q3)	$28611 ($12739-$60174)	$24947 ($9664-$51661)	$31642 ($13886-$63680)	$25223 ($9433-$55271)
Min – Max	$407 - $178345	$277 - $179460	$408 - $343468	$277 - $345853
Inpatient Rehabilitation Service				
Any – n (%)	3,710 (0.3%)	6,376 (0.5%)	5,505 (0.5%)	7,294 (0.6%)
Mean (SD)	$22652.0 ($16538.8)	$24009.5 ($15155.8)	$25825.1 ($20188.4)	$25194.2 ($17034.4)
Median (Q1-Q3)	$20777 ($12747-$26824)	$22357 ($16551-$26107)	$22357 ($16551-$29839)	$22357 ($16907-$27336)
Min – Max	$276 - $224538	$289 - $192495	$343 - $449075	$289 - $297881
Public Drug Plan				
Any – n (%)	443,029 (37.3%)	466,992 (39.3%)	533,621 (44.9%)	593,099 (49.9%)
Mean (SD)	$876.8 ($3959.6)	$811.1 ($3884.0)	$1411.1 ($6672.5)	$1222.2 ($6564.4)
Median (Q1-Q3)	$73 ($13-$675)	$64 ($22-$577)	$65 ($16-$906)	$55 ($20-$619)
Min – Max	$1 - $616775	$1 - $568013	$1 - $1145011	$1 - $1082077
Aggregated costs for other services^2^				
Any – n (%)	1,011,920 (85.1%)	1,029,490 (86.6%)	1,058,748 (89.0%)	1,081,160 (90.9%)
Mean (SD)	$835.0 ($6005.5)	$869.7 ($6041.4)	$1460.3 ($10644.8)	$1461.5($10394.9)
Median (Q1-Q3)	$114 ($50-$248)	$120 ($55-$296)	$218 ($92-$587)	$233 ($100-$633)
Min – Max	$1 - $348277	$1 - $348063	$1 - $655331	$1 - $576744

^1^ICU and Mechanical Ventilation Costs represented the inpatient costs for people with at least 1 ICU admission, or mechanical ventilation use, respectively (not the costs of the ICU stay and mechanical ventilation use).

^2^Aggregated cost for other services included direct costs for dialysis clinics, cancer clinic visits, chemotherapy, OHIP lab billings, OHIP non-physician billings, Family Health Organization/Family Health Network physician capitation, Ontario Mental Health Reporting System admissions, assisted devices, and outpatient hospital clinic visits.

Cost was estimated among the subpopulation among which who had at least one heath care touchpoint during the respective time period.

### Impact of COVID-19 vaccination

To understand the impact of COVID-19 vaccination on all-cause HCRU and associated costs, we examined the numerical differences in all-cause HCRU rates and associated costs from the look-back period to the follow-up period, across strata of vaccination status and time since last vaccine dose.

**Fig 1:**
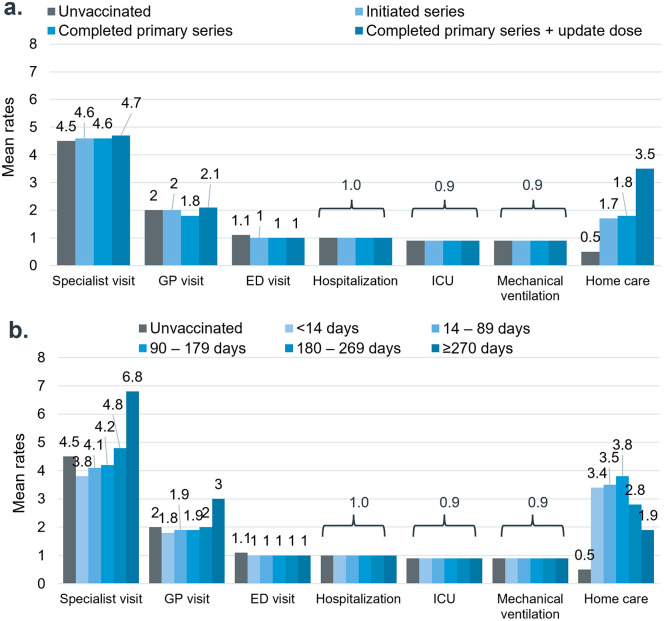
Increase in mean HCRU rate from look-back period to Month 1 by vaccination status. HCRU rates were evaluated for the entire study population, including participants with zero encounters within each HCRU category.

**Fig 2:**
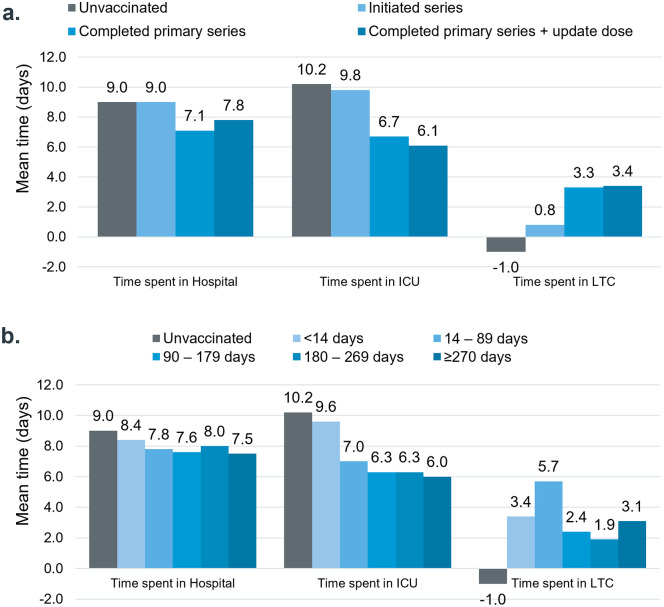
Increase in mean time spent in healthcare setting from look-back period to Month 1 by vaccination status. Time spent in healthcare settings was assessed for individuals who had at least one visit to the respective healthcare settings.

The pattern of increased all-cause HCRU in the first month following index COVID-19 infection compared to the preceding year was similar across groups regardless of vaccination status (Fig 1). However, vaccinated cases experienced a smaller increase in hospital and ICU days compared to unvaccinated cases, particularly among those with more complete vaccination. The average ICU duration increased by 10.2 days for unvaccinated cases and by 9.8 days for cases who had only initiated their COVID-19 vaccination series, while those who had completed the primary vaccination series and received an updated dose saw an increase of only 6.1 days (Fig 2). Similarly, vaccinated cases had a smaller increase in hospital days following index infection compared to the unvaccinated cases: unvaccinated cases and those who had only initiated their vaccination series showed an increase of 9 days, while cases who completed the primary series saw an increase of 7.1 days, and those who completed the primary series and received an updated dose had an increase of 7.8 days (Fig 2). COVID-19 patients given mRNA or non-mRNA vaccines had comparable rises in healthcare use from pre-index to one month after, but the mRNA group spent more days in the ICU (S1 Fig).

In terms of the timing of vaccination, the COVID-19 cases vaccinated within 6 months prior to infection showed a smaller incremental increase in the number of specialist visits, ranging from 3.8 to 4.8 visits PPPM in the preceding year to Month 1. In comparison, unvaccinated cases experienced an average increase of 4.5 visits PPPM, and those vaccinated more than 6 months prior to index experienced an average increase of 6.8 visits PPPM (Fig 1). Other HCRU categories did not exhibit a clear pattern of change following the index infection across groups, except for those vaccinated within 14 days prior to infection. This group showed a similar increase of 9.6 ICU days, comparable to unvaccinated cases who saw an increase of 10.2 ICU days, while other groups displayed a smaller increase ranging from 6 to 7 ICU days.

**Fig 3:**
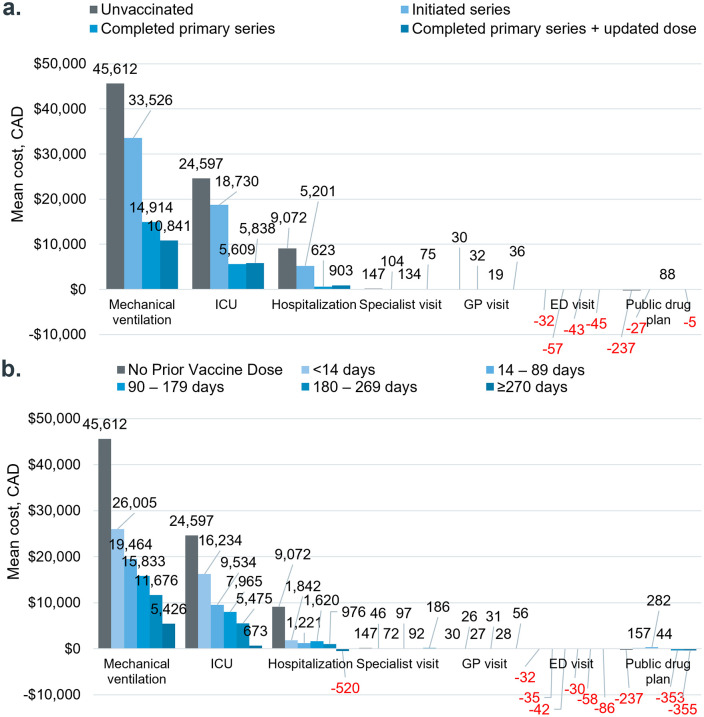
Increase in mean cost from 6 months pre- to post index, by vaccination status. Costs were analyzed for individual with at least one encounter to each HCRU type.

The increase in total mean per-person costs in the six months following index infection compared to the prior period was smaller in magnitude for cases with more complete vaccination status (Fig 3). This trend was also observed across healthcare resource categories, including costs associated with mechanical ventilation (unvaccinated: + $45,612; initiated series: + $33,526; completed primary series: + $14,914; completed primary series and updated dose: + $10,841), ICU stays (unvaccinated: + $24,597; initiated series: + $18,730; completed primary series: + $5,609; completed primary series and updated dose: + $5,838), and hospitalizations (unvaccinated: + $9,072; initiated series: + $5,201; completed primary series: + $623; completed primary series and updated dose: + $903) (Fig 3). The rise in costs for mechanical ventilation and ICU care from the look-back period to the follow-up period appeared attenuated among groups that had been vaccinated earlier. For example, cases vaccinated within 14 days prior to index had an increase of $16,234 in ICU admission expenses, while this incremental increase ranged from $9,534 for those vaccinated 14 days to 3 months before infection, to $5,475 for those vaccinated 6–9 months before infection (Fig 3).

## Discussion

This study examined all-cause HCRU and costs among approximately 1.3 million PCR-positive COVID-19 cases (i.e., people with SARS-CoV-2 positive PCR test) in Ontario, immediately following infection out to 12 months. We observed increased overall all-cause HCRU and costs following infection, with per-person-per-month rates rising 1–11 times across HCRU categories. The total costs to healthcare systems amounted to $5,873 (SD: $20,630.6) per person in the first 6 months, compared to $4,279 (SD: $15,670.9) in the 6-month look-back period. These findings are directionally consistent with phase‑based and survival‑adjusted Ontario estimates demonstrating persistent, post‑acute healthcare expenditures after SARS‑CoV‑2 infection, and with Canadian assessments of the post‑COVID condition burden on service utilization [14, 15, 16]. Differences in magnitude across studies likely reflect case‑mix, calendar time (2020 vs 2021–2023), and follow‑up windows, as well as our focus on PCR‑confirmed cases subject to evolving provincial eligibility criteria.

As previously reported, the increased all-cause HCRU and costs after COVID-19 infection were primarily attributable to ICU admission, mechanical ventilation use and hospitalizations. This pattern aligns with North American claims analyses showing concentrated inpatient costs during the first 30–60 days post‑diagnosis and high resource intensity among those requiring ICU care and mechanical ventilation [15, 17]. The study cohort reflected a geographic distribution similar to that of the general population [18]. However, the infected cohort was generally older and exhibited higher rates of comorbidities, such as chronic heart failure, chronic obstructive pulmonary disease, and dementia, consistent with existing literature on COVID-19 risk factors [19], and with broader U.S. county‑level analyses linking social vulnerability and healthcare access to worse outcomes over time [20]. Additionally, there was a higher representation of females in the infected group, likely due to the greater number of female healthcare workers who were more frequently tested by PCR testing methods.

Hospital admissions within the first month following index infection occurred in 6.7% of the study cohort compared to other findings of 5.5–5.9% within 60 days of infection [21], supporting the notion that the biggest impact on inpatient healthcare utilization occurs shortly after diagnosis of COVID-19. Our results suggested that 48.8% of ICU cases required mechanical ventilation. This observation adds to the growing body of literature from larger epidemiologic studies of COVID-19. For example, rates of invasive mechanical ventilation among ICU cases have been reported to range from 29.1% in one study from China [22] to 89.9% in a U.S. study [23] and anywhere from 2.3% to 33.1% among hospitalized patients [24]. From a preparedness standpoint, these mechanical ventilation and ICU costs underscore the importance of maintaining surge‑ready critical care capacity, consistent with cross‑national work suggesting that ventilator availability and robust acute‑care infrastructure are associated with lower fatality risk during respiratory pandemics [25, 26].

The observed increase in GP and specialist visits following COVID‑19 infection should be interpreted with caution. While this pattern may partly reflect sequelae of the infection, it is also likely influenced by heightened medical surveillance and routine follow‑up care.

Our findings suggest that vaccination may provide a protective effect against the increased all-cause HCRU and healthcare costs associated with COVID infection by reducing the need for mechanical ventilation, ICU stays, hospitalization rates, and overall hospital days. This aligns with recent international and Canadian evidence [27, 28, 29, 30]. national surveillance analyses show substantially lower risks of hospitalization and death among vaccinated individuals compared with unvaccinated persons, and recent cost‑utility models indicate that seasonal, risk‑based vaccination strategies (e.g., annual for adults ≥65 years and biannual for the highest‑risk groups) are cost‑effective in the endemic period [31, 32]. These findings are consistent with current NACI guidance recommending annual COVID‑19 vaccination during the respiratory season to reduce severe outcomes and preserve health‑system capacity [33].

Methodologically, this study assessed the impact of vaccination by comparing mean differences in all-cause HCRU and costs post- versus pre-index COVID-19 infection, by vaccination status. The use of a pre-post comparison allowed for a within-groups comparison, with the benefit of minimizing the impact of confounding factors such as age, comorbidities, and sex. However, it should be noted that only numerical differences in HCRU and costs before and after infection were examined; no statistical comparisons were made so causes for cost variation weren’t established. Exploratory analyses showed that costs within six months post-infection were highest among individuals with three or more vaccine doses, likely reflecting higher prevalence of comorbidities in this group. For example, subgroups with three or more doses had the highest prevalence of chronic heart disease (3 doses: 14.8%; 1 dose: 7%; 2 doses: 4.6%; 0 dose: 4%). These results suggest comorbidities contribute to observed cost differences between vaccinated and unvaccinated people. Caution is advised in interpretation. Vaccination status was treated as a fixed exposure defined at index and changes in vaccination status following the index infection were not accounted. Future studies could build on these findings by including statistical comparisons and modeling vaccination as a time‑varying exposure to further characterize incremental burden associated with COVID-19 infection by vaccination status.

This study has several limitations. First, this study does not exclude cases where hospitalization occurred on the same day as a positive PCR test, such as when PCR testing is routine during hospital admissions, which may skew HCRU, particularly hospitalizations, to appear more frequent in the first month following index. This study assessed all-cause HCRU, which may limit identifying COVID-19-specific signals. Previous research shows accurately coding true COVID-19 admissions with administrative data is difficult, as many patients are miscoded or unconfirmed [34]. Therefore, using all-cause hospitalization provides a consistent, though broad, outcome measure. Second, the cohort was restricted to PCR-confirmed infections, influenced by provincial testing criteria, which may affect generalisability. ICES data also excludes private and cash claims, and measurement errors typical of administrative databases remain possible despite validated algorithms. Third, modelling choices have constraints. Poisson, Negative Binomial, and Gamma models may be sensitive to outliers; contaminated versions could be considered in future work. While over-dispersion checks were performed and a self-matched design used to control for confounders, unadjusted models and repeated measures may introduce bias. More robust approaches such as GEE or mixed-effects models are noted for future research. Lastly, the study analyzed data from January 1, 2021, to May 31, 2023, spanning both the pre-Omicron and Omicron-predominant eras. It is possible that variations in virus characteristics and case severity across this period may skew results, depending on the specific period that cases were indexed. Future analyses may aim to stratify analyses of disease burden by variant periods, align analyses with seasonal vaccination timing, and conduct sensitivity analyses excluding same‑day hospital PCR to address potential index‑date misclassification [14, 33].

## Conclusion

This study described increased all-cause HCRU among people with a PCR-positive test for SARS-CoV-2. These data indicate that COVID-19 is associated with high use of mechanical ventilation, ICU admissions, hospitalizations, and physician visits, which could potentially be alleviated by vaccination. From a system perspective, the concentration of costs in critical care episodes highlights the need to preserve ICU/mechanical ventilation surge capacity and to plan for post‑acute/PCC services, while integrating equity‑sensitive metrics (e.g., neighborhood deprivation) to anticipate HCRU and costs in populations with higher social vulnerability [16], [20], [26]. These insights can inform patient counselling to address vaccine hesitancy, guide public information campaigns, and support resource planning as Ontario navigates the lingering effects of the pandemic and transitions to seasonal immunization strategies.

## Supporting information

S1 Table Attrition Table. (DOCX)

S2 Table Description of health administrative databases held at ICES used in the study. (DOCX)

S3 Table Variable operational definitions. (DOCX)

S1 File Variables for stratified analysis. (DOCX)

S2 File Statistical comparison of mean HCRU rates and mean costs between lookback period and analysis period. (DOCX)

S1 Fig Increase in mean HCRU rate and mean time spent in healthcare setting from look-back period to Month 1 by type of vaccine. (TIF)
