# Supplementary material for: Healthcare resource utilization and cost burden of COVID-19 according to vaccination status in adults in Ontario, Canada, 2021–2023
Source: PLoS One. 2026 Apr 22;21(4):e0344690. doi: 10.1371/journal.pone.0344690 (PMC13102196; doi:10.1371/journal.pone.0344690)
Supplement: S1 Table — (DOCX) [file pone.0344690.s001.docx]

Supplementary File 1: Attrition Table

|  | **Cohort Selection Criteria** | **Excluded** | **Remaining** |
| --- | --- | --- | --- |
| ***Cases identified on the first positive PCR test* for SARS-CoV-2 during selection period (i.e., Jan 1, 2021 to May 31, 2023) based on the case definition*** | |  | 1,354,651 |
| **Inclusion Criteria** | Age ≥6 months at index date | 5,945 | 1,348,706 |
| **Exclusion Criteria** |  |  |  |
|  | Invalid OHIP card number or not eligible for OHIP on index date | 21,836 | 1,326,870 |
|  | Invalid or incomplete records (e.g., missing age, missing sex, death before index date) | 679 | 1,326,191 |
|  | Non-Ontario resident on index date | 1,414 | 1,324,777 |
|  | Age ≥105 years at index date | 129 | 1,324,648 |
|  | OHIP eligible for <1 month pre-index date | 3,474 | 1,321,174 |
| **HCRU cohort** |  |  | 1,321,174 |
|  | Cases with an index date from January 1, 2021, to September 30, 2022 | 132,169 | 1,189,005 (90.0%) |
| **Direct cost cohort** |  |  | 1,189,005 |
