# Supplementary material for: Healthcare resource utilization and cost burden of COVID-19 according to vaccination status in adults in Ontario, Canada, 2021–2023
Source: PLoS One. 2026 Apr 22;21(4):e0344690. doi: 10.1371/journal.pone.0344690 (PMC13102196; doi:10.1371/journal.pone.0344690)
Supplement: S2 Table — (DOCX) [file pone.0344690.s002.docx]

Supplementary File 2: Description of health administrative databases held at ICES used in the study

| **Name** | **Description** | **Examples of variables collected** |
| --- | --- | --- |
| Discharge Abstract Database (DAD) | Administrative, clinical, and demographic information on hospital discharges (including deaths, sign-outs and transfers) | Inpatient hospitalization |
| National Ambulatory Care Reporting System (NACRS) | Captures information on ambulatory care visits to emergency departments, outpatient and community-based clinics, and day surgeries | ED visits |
| Ontario Health Insurance Plan (OHIP) | Captures most claims paid for by OHIP, which provides information on the type of service provided. Approximately 94% of Ontario physicians have a fee-for-service practice billed almost exclusively to OHIP | Physician billings |
| Postal Code Conversion File (PCCF) | Links to postal codes within a given cohort and determine other census geographic identifiers such as neighbourhood income quintile | Income quintile |
| Registered Persons Database (RPDB) | Demographic information on all individuals with an Ontario health card (OHIP card) | Age, sex, rurality, LHIN, date of death |
| Home Care Database (HCD) | Captures all services provided by or coordinated by Ontario’s Community Care Access Centres (CCACs) | Long-term care residency |
| Ontario Laboratories Information System, COVID-19 Laboratory Data (OLISC19) | A subset of OLIS information repository that contains lab test orders and results from hospitals, community labs and public health labs specific to COVID-19. The data feed does not include records with a consent block in place. | PCR tests for SARS-CoV-2 |
| Ontario COVID-19 Vaccine Data (COVAXON) | Central data repository for COVID-19 vaccine data and reporting in Ontario, administered by the Ontario Ministry of Health. | COVID-19 vaccine status |
| Ontario Drug Benefit (ODB) | Database of medications reimbursed by the Government of Ontario | Drug claims |
| COVID19 Integrated Testing Data C19INTGR (OLIS, DL, CCM) | Captures available COVID-19 diagnostic laboratory results in Ontario. | Positive COVID-19 test |
| National Rehabilitation Reporting System (NRS) | Patient-level demographic, diagnostic, procedural and treatment information from participating adult inpatient rehabilitation facilities and programs | Inpatient rehabilitation services |
| Continuing Care Reporting System-Long term care (CCRS-LTC) | Admission of the individual in the Continuing Care Reporting System’s database | Long-term care |
| Same Day Surgery Database (SDS) | Patient-level demographic, diagnostic, procedural and treatment information on all day surgeries | Surgeries |
| Ontario Diabetes Dataset (ODD) | Registry of Ontario residents diagnosed with diabetes | Diabetes |
| Ontario Asthma Dataset (ASTHMA) | Contains all Ontario asthma patients identified since 1991 | Asthma |
| Ontario Crohn’s and Colitis Cohort Dataset (OCCC) | Cohort of pediatric-onset (six months to 18 years) inflammatory bowel disease patients derived from health administrative data | Inflammatory bowel disease |
| Ontario HIV Database (HIV) | Administrative registry of individuals with diagnosed HIV | HIV |
