# Supplementary material for: Healthcare resource utilization and cost burden of COVID-19 according to vaccination status in adults in Ontario, Canada, 2021–2023
Source: PLoS One. 2026 Apr 22;21(4):e0344690. doi: 10.1371/journal.pone.0344690 (PMC13102196; doi:10.1371/journal.pone.0344690)
Supplement: S3 Table — (DOCX) [file pone.0344690.s003.docx]

Supplementary File 3: Variable operational definitions

| **Variable** | **Time Period** | **Data source** | **Operational definition** |
| --- | --- | --- | --- |
| **Demographic characteristics** |  |  |  |
| **Age**  (Years, 0.5 years to 104 years (Continuous);  0.5 to < 5 years, 5 to <12 years, 12 to <18 years, 18 to <65 years, ≥65 years; <18 years, ≥18 years (Categorical)) | Index date | RPDB | Continuous, Categorial |
| **Sex**  (Male, female) | Up to 12-month lookback | RPDB | Categorical |
| **Rurality**  (Large urban, medium urban, rural) | Up to 12-month lookback | RPDB | Categorical |
| **Local Health Integration Network (LHIN)**  (Erie St. Clair, South West, Waterloo Wellington, Hamilton Niagara Haldimand Brant, Central West, Mississauga Halton, Toronto Central, Central, Central East, South East, Champlain, North Simcoe Muskoka, North East, North West) | Up to 12-month lookback | RPDB | Categorical |
| **Neighbourhood income quintile**  (Q1 – lowest, Q2, Q3, Q4, Q5 – highest) | Up to 12-month lookback | RPDB | Ordinal |
| **Long-term care residency**  (Yes, No) | Up to 12-month lookback | ODB, OHIP, CCRS_LTC | Binary |
| **Clinical characteristics** |  |  |  |
| **Charlson Comorbidity Index** (i.e., only for in-hospital patients)  (0, 1, 2, 3+, Missing) | Up to 12-month lookback | DAD | Categorical |
| **Specific comorbidities** (e.g., diabetes, chronic pulmonary disease, cardiovascular disease, immunocompromised)  (Yes, No) | Up to April 1, 2002 | ICES-derived/-acquired cohorts or DAD, NACRS, OHIP, SDS | Binary |
| **Other respiratory infections** (i.e., respiratory syncytial virus (RSV), influenza (flu))  (Yes, No) | Up to 12-month lookback; up to 12-month analysis period | DAD, NACRS, OHIP, SDS | Binary |
| **COVID-19 vaccination status**  (Yes, No; 0, 1, 2, or >3) | Up to April 1, 2002 | COVAXON | Binary, Categorical |
| **Type of COVID-19 vaccine(s)**  (mRNA, non-mRNA, combination of mRNA and non-mRNA, none) | Up to April 1, 2002 | COVAXON | Categorical |
| **Time from last COVID-19 vaccine dose**  (<14. 14 – 89, 90 – 179, 180 – 269, or ≥270 days, no prior vaccine dose) | Up to April 1, 2002 | COVAXON | Categorical |
| **Time since last positive PCR test for SARS-CoV-2**  (<14. 14 – 89, 90 – 179, 180 – 269, or ≥270 days, no prior positive PCR test) | Up to April 1, 2002 | OLIS | Categorical |
| **All-cause HCRU** |  |  |  |
| **Outpatient visits**  (Number of visits) | Analysis Period | OHIP | Continuous |
| **Primary care visits**  (Number of visits) | Analysis Period | OHIP | Continuous |
| **Hospitalizations**  (Number of hospitalizations) | Analysis Period | DAD | Continuous |
| **ED visits**  (Number of visits) | Analysis Period | NACRS | Continuous |
| **Length of stay in hospital**  (Total number of days) | Analysis Period | DAD | Continuous |
| **ICU admission**  (Yes, No) | Analysis Period | DAD | Binary |
| **ICU admission**  (Number of visits) | Analysis Period | DAD | Continuous |
| **Time in ICU**  (Total number of days) | Analysis Period | DAD | Continuous |
| **Mechanical ventilation**  (Yes, No) | Analysis Period | DAD | Binary |
| **Same day surgeries**  (Number of surgeries) | Analysis Period | SDS | Continuous |
| **Long term care**  (Total number of days) | Analysis Period | HCD | Continuous |
| **Long term care**  (Number of visits) | Analysis Period | HCD | Continuous |
| **Home care services**  (Number of visits) | Analysis Period | HCD | Continuous |
| **Inpatient rehabilitation services**  (Total number of days) | Analysis Period | NRS | Continuous |
| **Healthcare Costs** |  |  |  |
| **Total costs and by heath care categories**  (e.g., physician services, ED, hospitalizations, ICU admission, mechanical ventilation, same-day surgery, long-term care, home care, complex continuing care, inpatient rehabilitation, other, drugs) | Analysis Period | Various databases via ICES Costing Algorithm | Continuous |
| **Public drug plan costs**  (Dollar figure) | Analysis Period | ODB | Continuous |
