# Supplementary material for: Healthcare resource utilization and cost burden of COVID-19 according to vaccination status in adults in Ontario, Canada, 2021–2023
Source: PLoS One. 2026 Apr 22;21(4):e0344690. doi: 10.1371/journal.pone.0344690 (PMC13102196; doi:10.1371/journal.pone.0344690)
Supplement: S1 File — (DOCX) [file pone.0344690.s004.docx]

Supplementary File 4: Variables for stratified analysis

**COVID-19 vaccination status**

The number of doses of SARS-CoV-2 vaccines (i.e., 0, 1, 2, or ≥3) received prior to the index date was captured using the Ontario COVID-19 Vaccine Data COVaxON dataset.

In addition, the completion of Primary Series was captured using the following definitions:

1. Initiated Primary Series

Refers to all individuals who have received one dose of a two-dose vaccine series of any COVID-19 vaccine, whether it is authorized by Health Canada (Pfizer-BioNTech, Moderna, Astrazeneca/Covishield, Novavax, or Medicago) or authorized by World Health Organization (WHO) (Serum Institute of India, Bharat Biotech/Covaxin, Coronavac/Sinovac or Sinopharm/BBIBP) or not

1. Completed Primary Series

Refers to individuals who have any of the following:

1. Two doses of COVID-19 vaccines authorized by Health Canada or authorized by WHO including any mixed combination, OR
2. One dose of Johnson & Johnson’s Janssen or CanSinoBio's Convidecia COVID-19 vaccine, OR
3. One dose of a WHO authorized COVID-19 vaccine AND one dose of a Health Canada authorized COVID-19 vaccine (regardless of the order), OR
4. Three doses of a non-Health Canada authorized vaccine product, where the date of the primary series completion would be the second dose if the first two doses were WHO authorized, OR
5. One dose of a Health Canada authorized COVID-19 vaccine AND two doses of a non-Health Canada authorized COVID-19 vaccine (regardless of the order)
   1. Exception: If first two doses were WHO-approved, the following dose of Health Canada authorized vaccine would classify as a updated dose. The individual would meet criteria for “Completed Primary Series + Updated dose”.
6. Completed Primary Series + updated dose

Refers to individuals who have met the completed primary series criteria AND have received an additional one to three doses of a COVID-19 vaccine authorized by Health Canada as a updated dose (Pfizer-BioNTech or Moderna)

Please note, that 1) number of doses of SARS-CoV-2 vaccines (i.e., 0, 1, 2, or ≥3) 2) completion of Primary Series (i.e., categories 1 to 3) are mutually exclusive.

**Time since last vaccine dose**

The number of days between the last vaccine dose and the index date (i.e., <14, 14 – 89, 90 – 179, 180 – 269, ≥270 days, no prior vaccine dose) was calculated using data from the COVAXON dataset. A database history lookback period prior to the index date was used for calculation. The start of the database history lookback period was set at the earliest date available in the database for COVID-19 vaccines (i.e., December 2020). The index date was excluded from the lookback window.
