# Supplementary material for: Healthcare resource utilization and cost burden of COVID-19 according to vaccination status in adults in Ontario, Canada, 2021–2023
Source: PLoS One. 2026 Apr 22;21(4):e0344690. doi: 10.1371/journal.pone.0344690 (PMC13102196; doi:10.1371/journal.pone.0344690)
Supplement: S2 File — (DOCX) [file pone.0344690.s005.docx]

**Supplementary File 5: Statistical comparison of mean HCRU rates and mean costs between lookback period and analysis period**

Table 1 Comparison of mean HCRU rates between Lookback Period and Months 1-3, HCRU cohort

|  |  | **Lookback Period (PPPM) vs Month 1** | | | | | **Lookback Period (PPPM) vs Month 2** | | | | | **Lookback Period (PPPM) vs Month 3** | | | | |
| --- | --- | --- | --- | --- | --- | --- | --- | --- | --- | --- | --- | --- | --- | --- | --- | --- |
|  | **Statistical Measure** | **Estimate** | **Standard Error** | **95% Lower** | **95% Upper** | **p-value** | **Estimate** | **Standard Error** | **95% Lower** | **95% Upper** | **p-value** | **Estimate** | **Standard Error** | **95% Lower** | **95% Upper** | **p-value** |
| **GP visits at any settings** | Rate Ratio | 2.40 | 0.00 | 2.39 | 2.41 | <.0001 | 1.33 | 0.00 | 1.33 | 1.34 | <.0001 | 1.14 | 0.00 | 1.14 | 1.15 | <.0001 |
|  | Rate Difference | 73.24 | 0.00 | 72.80 | 73.68 | <.0001 | 17.28 | 0.00 | 16.96 | 17.60 | <.0001 | 7.37 | 0.00 | 7.10 | 7.64 | <.0001 |
| **Specialist visits at any settings** | Rate Ratio | 3.02 | 0.00 | 3.00 | 3.03 | <.0001 | 1.41 | 0.00 | 1.40 | 1.42 | <.0001 | 1.16 | 0.00 | 1.16 | 1.17 | <.0001 |
|  | Rate Difference | 121.62 | 0.01 | 120.62 | 122.63 | <.0001 | 25.00 | 0.00 | 24.39 | 25.60 | <.0001 | 9.89 | 0.00 | 9.39 | 10.39 | <.0001 |
| **Emergency department visits** | Rate Ratio | 3.09 | 0.00 | 3.07 | 3.11 | <.0001 | 1.00 | 0.00 | 0.99 | 1.01 | 0.9324 | 0.95 | 0.00 | 0.94 | 0.96 | <.0001 |
|  | Rate Difference | 10.92 | 0.00 | 10.85 | 11.00 | <.0001 | 0.00 | 0.00 | -0.05 | 0.05 | 0.9324 | -0.25 | 0.00 | -0.30 | -0.20 | <.0001 |
| **Inpatient hospitalizations** | Rate Ratio | 5.87 | 0.00 | 5.82 | 5.92 | <.0001 | 1.21 | 0.01 | 1.19 | 1.23 | <.0001 | 1.05 | 0.01 | 1.03 | 1.07 | <.0001 |
|  | Rate Difference | 6.03 | 0.00 | 5.98 | 6.08 | <.0001 | 0.26 | 0.00 | 0.24 | 0.28 | <.0001 | 0.06 | 0.00 | 0.04 | 0.08 | <.0001 |
| **ICU admission** | Rate Ratio | 8.14 | 0.01 | 7.97 | 8.31 | <.0001 | 1.14 | 0.02 | 1.09 | 1.19 | <.0001 | 0.92 | 0.02 | 0.88 | 0.97 | 0.0013 |
|  | Rate Difference | 1.06 | 0.00 | 1.04 | 1.08 | <.0001 | 0.02 | 0.00 | 0.01 | 0.03 | <.0001 | -0.01 | 0.00 | -0.02 | 0.00 | 0.0009 |
| **Mechanical ventilation use** | Rate Ratio | 11.91 | 0.02 | 11.55 | 12.29 | <.0001 | 1.34 | 0.04 | 1.25 | 1.44 | <.0001 | 1.02 | 0.04 | 0.94 | 1.11 | 0.5991 |
|  | Rate Difference | 0.54 | 0.00 | 0.52 | 0.55 | <.0001 | 0.02 | 0.00 | 0.01 | 0.02 | <.0001 | 0.00 | 0.00 | 0.00 | 0.01 | 0.6024 |
| **Same-day surgery** | Rate Ratio | 1.08 | 0.01 | 1.06 | 1.10 | <.0001 | 1.28 | 0.01 | 1.26 | 1.31 | <.0001 | 1.18 | 0.01 | 1.15 | 1.20 | <.0001 |
|  | Rate Difference | 0.06 | 0.00 | 0.05 | 0.08 | <.0001 | 0.23 | 0.00 | 0.21 | 0.25 | <.0001 | 0.14 | 0.00 | 0.12 | 0.16 | <.0001 |
| **Home care services** | Rate Ratio | 0.96 | 0.00 | 0.95 | 0.96 | <.0001 | 1.09 | 0.00 | 1.08 | 1.10 | <.0001 | 1.06 | 0.00 | 1.05 | 1.07 | <.0001 |
|  | Rate Difference | -2.45 | 0.00 | -2.88 | -2.03 | <.0001 | 4.98 | 0.00 | 4.49 | 5.47 | <.0001 | 3.35 | 0.00 | 2.85 | 3.86 | <.0001 |
| **Inpatient rehabilitation services** | Rate Ratio | 6.03 | 0.02 | 5.81 | 6.26 | <.0001 | 1.98 | 0.03 | 1.87 | 2.10 | <.0001 | 1.04 | 0.04 | 0.96 | 1.12 | 0.3002 |
|  | Rate Difference | 0.27 | 0.00 | 0.26 | 0.28 | <.0001 | 0.05 | 0.00 | 0.05 | 0.06 | <.0001 | 0.00 | 0.00 | 0.00 | 0.01 | 0.3084 |
| **Any HCRU (listed above)** | Rate Ratio | 2.21 | 0.00 | 2.20 | 2.22 | <.0001 | 1.27 | 0.00 | 1.27 | 1.28 | <.0001 | 1.12 | 0.00 | 1.11 | 1.12 | <.0001 |
|  | Rate Difference | 211.57 | 0.01 | 210.25 | 212.90 | <.0001 | 47.82 | 0.00 | 46.90 | 48.74 | <.0001 | 20.54 | 0.00 | 19.70 | 21.38 | <.0001 |

Note: The statistical estimates of rate ratio and rate difference between respective time periods were calculated using generalized estimating equation methodology. The mean number of HCRU touchpoints during the respective time periods were modeled using the log link function.

Table 2 Comparison of costs between Lookback Period and Analysis Period, Direct cost cohort

|  | **6 Months before index date vs. 6 months after index date** | | | | | |
| --- | --- | --- | --- | --- | --- | --- |
|  | **Statistical Measure** | **Estimate** | **Standard Error** | **95% Lower** | **95% Upper** | **p-value** |
| **GP visits at any settings** | Relative Mean Difference | 1.24 | 0.00 | 1.23 | 1.24 | <.0001 |
|  | Absolute Mean Difference | 29.69 | 0.36 | 28.99 | 30.40 | <.0001 |
| **Specialist visits at any settings** | Relative Mean Difference | 1.27 | 0.00 | 1.27 | 1.28 | <.0001 |
|  | Absolute Mean Difference | 102.37 | 1.20 | 100.01 | 104.73 | <.0001 |
| **Emergency department visits** | Relative Mean Difference | 1.11 | 0.00 | 1.10 | 1.11 | <.0001 |
|  | Absolute Mean Difference | 16.17 | 0.50 | 15.19 | 17.15 | <.0001 |
| **Inpatient hospitalization** | Relative Mean Difference | 1.00 | 0.00 | 1.00 | 1.00 | <.0001 |
|  | Absolute Mean Difference | 0.00 | 0.00 | 0.00 | 0.00 | <.0001 |
| **ICU^1^** | Relative Mean Difference | 1.00 | 0.00 | 1.00 | 1.00 | <.0001 |
|  | Absolute Mean Difference | 0.00 | 0.00 | 0.00 | 0.00 | <.0001 |
| **Mechanical ventilation use^1^** | Relative Mean Difference | 1.00 | 0.00 | 1.00 | 1.00 | <.0001 |
|  | Absolute Mean Difference | 0.00 | 0.00 | 0.00 | 0.00 | <.0001 |
| **Same-day surgery** | Relative Mean Difference | 1.11 | 0.00 | 1.10 | 1.12 | <.0001 |
|  | Absolute Mean Difference | 7.96 | 0.24 | 7.48 | 8.43 | <.0001 |
| **Long-term care** | Relative Mean Difference | 1.00 | 0.00 | 1.00 | 1.00 | <.0001 |
|  | Absolute Mean Difference | 0.00 | 0.00 | 0.00 | 0.00 | <.0001 |
| **Home care** | Relative Mean Difference | 1.00 | 0.00 | 1.00 | 1.00 | <.0001 |
|  | Absolute Mean Difference | 0.00 | 0.00 | 0.00 | 0.00 | <.0001 |
| **Complex continuing care** | Relative Mean Difference | 1.00 | 0.00 | 1.00 | 1.00 | <.0001 |
|  | Absolute Mean Difference | 0.00 | 0.00 | 0.00 | 0.00 | <.0001 |
| **Inpatient rehabilitation** | Relative Mean Difference | 0.99 | 0.00 | 0.99 | 0.99 | <.0001 |
|  | Absolute Mean Difference | -0.01 | 0.00 | -0.01 | -0.01 | <.0001 |
| **Public drug plan** | Relative Mean Difference | 0.98 | 0.00 | 0.97 | 0.98 | <.0001 |
|  | Absolute Mean Difference | -8.17 | 1.01 | -10.15 | -6.18 | <.0001 |
| **Aggregated costs for other services^2^** | Relative Mean Difference | 1.06 | 0.00 | 1.05 | 1.06 | <.0001 |
|  | Absolute Mean Difference | 42.36 | 1.80 | 38.83 | 45.89 | <.0001 |
| **Total costs excluding public drug plan** | Relative Mean Difference | 1.44 | 0.00 | 1.43 | 1.45 | <.0001 |
|  | Absolute Mean Difference | 1617.53 | 11.73 | 1594.53 | 1640.53 | <.0001 |
| **Total costs** | Relative Mean Difference | 1.40 | 0.00 | 1.39 | 1.41 | <.0001 |
|  | Absolute Mean Difference | 1609.38 | 12.36 | 1585.15 | 1633.60 | <.0001 |

Note: The statistical estimates of relative mean difference and absolute mean difference of costs between respective time periods were calculated using generalized estimating equation methodology. The mean costs during the respective time periods were modeled using the log link function or power(-1) link function, where appropriate. Specifically, the power(-1) link function was used for estimates of costs for inpatient hospitalization, ICU, mechanical ventilation use, long-term care, home care, complex continuing care, and inpatient rehabilitation as the gamma distribution model with a log link function did not converge.

1 ICU and Mechanical Ventilation Costs represented the inpatient costs for people with at least 1 ICU admission, or mechanical ventilation use, respectively (not the costs of the ICU stay and mechanical ventilation use).

2 Aggregated cost for other services included direct costs for dialysis clinics, cancer clinic visits, chemotherapy, OHIP lab billings, OHIP non-physician billings, FHO/FHN physician capitation, OMHRS admissions, assisted devices, and outpatient hospital clinic visits
