# Supplementary figures and images for: Healthcare resource utilization and cost burden of COVID-19 according to vaccination status in adults in Ontario, Canada, 2021–2023
Source: PLoS One. 2026 Apr 22;21(4):e0344690. doi: 10.1371/journal.pone.0344690 (PMC13102196; doi:10.1371/journal.pone.0344690)

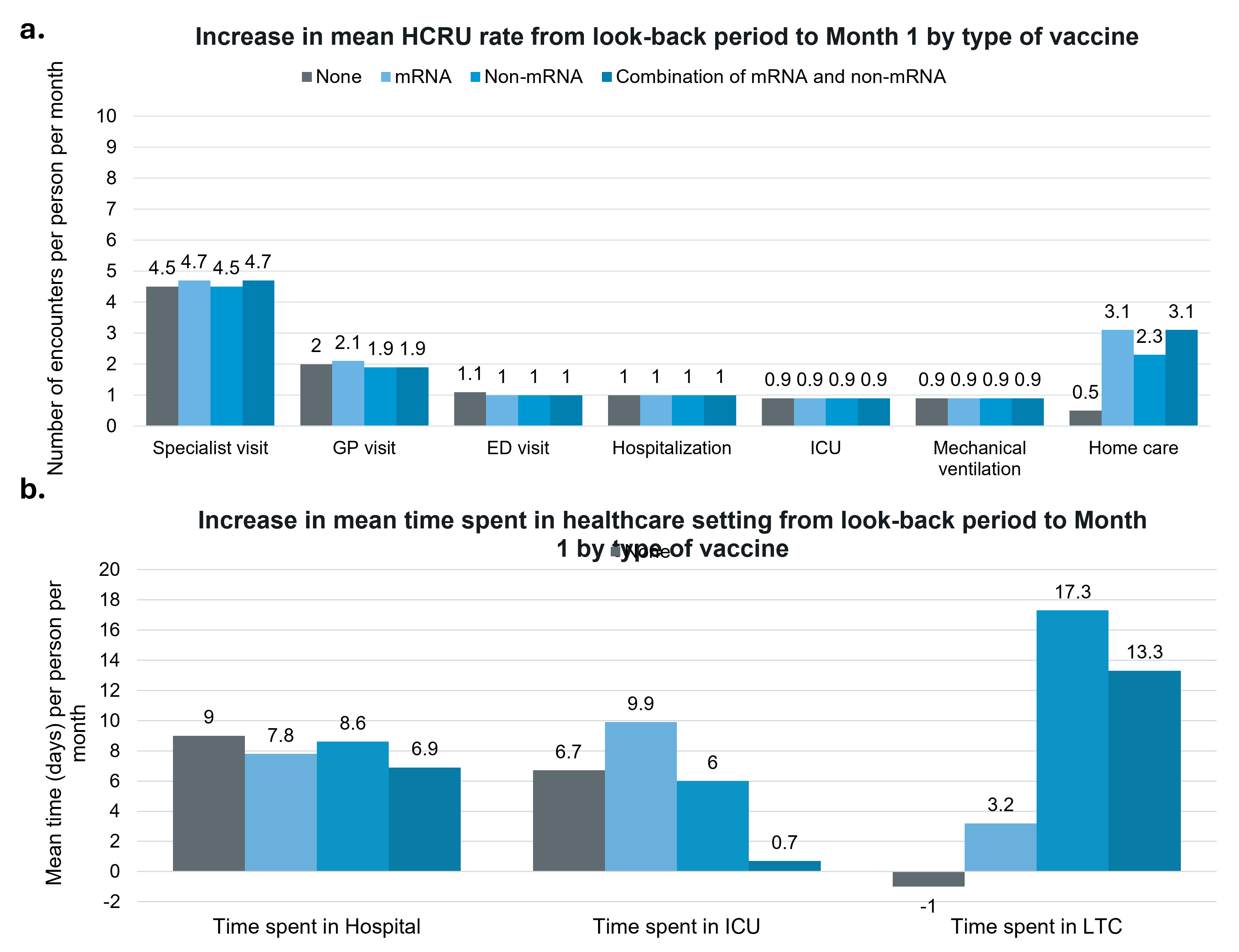

Supplement: S1 Fig — (TIF) [file pone.0344690.s006.tif]
